# Multiple hallucinations due to brainstem injury: A case report

**DOI:** 10.1590/S1980-57642010DN40400016

**Published:** 2010

**Authors:** Melissa Castello Branco e Silva, Sonia Maria Dozzi Brucki

**Affiliations:** 1 Behavioral and Cognitive Neurology Unit, Department of Neurology, University of São Paulo School of Medicine, São Paulo SP, Brazil.

**Keywords:** auditory hallucinations, visual hallucinations, brainstem encephalitis

## Abstract

We report a case of a 43-year-old woman with brainstem encephalitis in the third trimester of pregnancy. She presented complex visual and auditory hallucinations in the acute disease phase (hearing opera arias and seeing room furniture upside-down). Hallucinations resolved with antiviral treatment.

## Abstract (pt)

Caso de uma mulher de 43 anos, gestante, que apresentou alucinações visuais e auditivas (árias de ópera e visão de móveis do quarto de cabeça para baixo) associadas a fase aguda de encefalite de tronco cerebral. Os sintomas desapareceram com resolução do quadro.

Hallucinations can be defined as any sensory experience that occur without stimuli, and are classified as tactile, auditory (AH) or visual (VH). Hallucinations may be elementary (e.g. hearing single sounds or seeing lights) or complex, when music or voices or a scene are perceived in the absence of eliciting stimuli. The patient may or may not be fully aware of their imaginary nature.^1-3^

We report the case of a patient with bilateral auditory hallucinations and a short episode of visual hallucinations due to a brainstem injury.

## Case report

A 43-year-old woman at the 31^st^ week of pregnancy awoke in the middle of the night with a severe headache and tingling on the right side of her face and scalp, dizziness and nausea, followed by diplopia, rhinolalia, gait imbalance and loss of taste perception. She had had been treated for a ductal breast carcinoma four years earlier, with chemotherapy and quadrantectomy. Cancer was deemed under control.

On hospital admission two days later, neurological examination showed an alert and communicative patient with dysarthria, nasal voice, bilateral palate and tongue palsy, horizontal paresis of the right eye in both directions, and paresis of left eye to the left side left peripheral facial paresis, tactile and pain facial hypoesthesia in right trigeminal (V1, V2 and V3) territories, as well as at higher right cervical roots (C1, C2 and C3); right arm dysmetria, and gait ataxia. She progressed to four limb paresis over the subsequent two days.

Glucose, CBC, Sodium, Potassium, urea, creatinine, TSH, AST, ALT, albumin were all normal. HIV, *Borrelia burgdorferi, Listeria monocytogenes Toxoplasma gondii*, and varicella zoster serum antibodies were negative. CSF examination, obtained on the sixth disease day, showed 6 cells, glucose of 50 mg/dl and protein of 23 mg/dl. Acyclovir 10 mg/kg/day and Solumedrol 1 mg/kg/day for three days were started.

**Figure 1:**
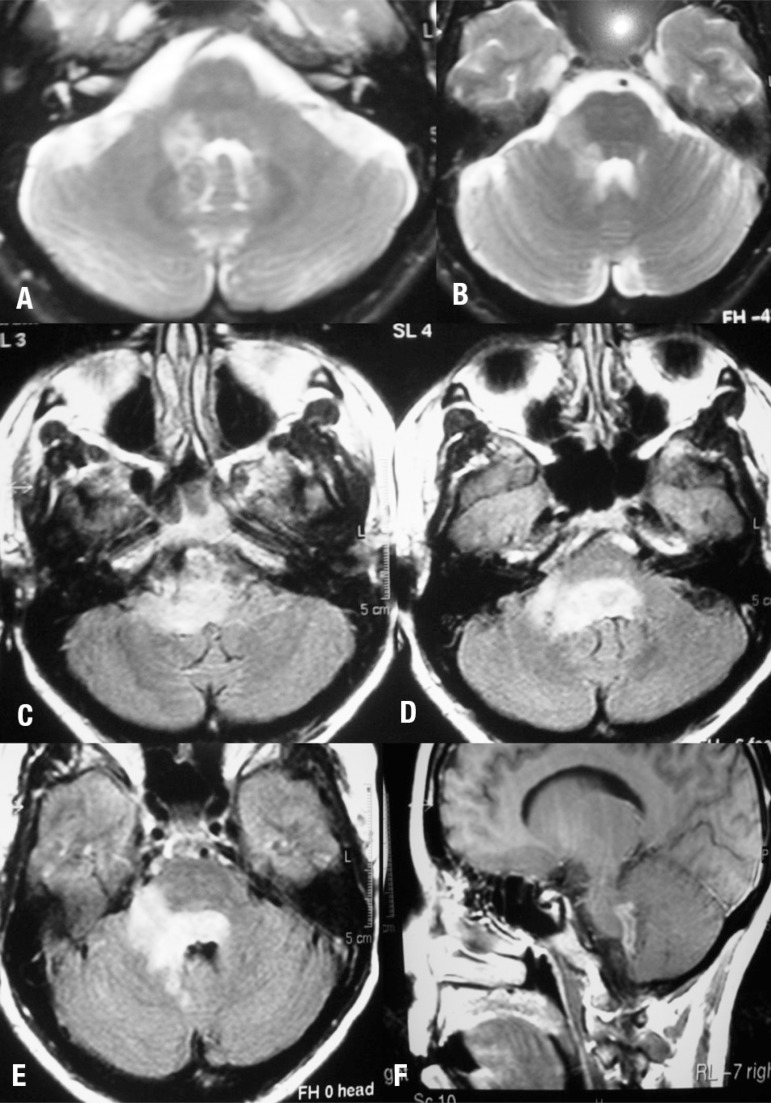
MRI- T2-weighted images [A, B], FLAIR [C, D, and E], T1 with gadolinium enhancement [F].

A brain MRI showed hyperintense signal on T2-weighted images in the right cerebellar peduncle and in the pons (Figure 1A and 1B). Acyclovir was then corrected to 30 mg/kg/day. After three days she presented uncontrollable vomiting. A repeat MRI showed progression of the lesions (Figure 1C, 1D, 1E, and 1F). The following day the patient underwent an emergency cesarean section due to fetal distress. Her clinical condition deteriorated, with septic shock secondary to pulmonary infection, requiring tracheal intubation. The antibiotic regimen was broadened with Imipenem and Vancomycin.

On the 8^th^ antiviral therapy day, CSF exam was repeated and showed 230 cells (92% lymphocytes), protein 59 mg/dll, glucose 57 mg/dl, negative herpes simplex virus polymerase chain reaction (PCR) test, positive HSV IGG titers (3.34) and negative cytology for neoplastic cells.

Patient had a favorable outcome, and was extubated. While still in the ICU, she presented two episodes of upside-down vision that lasted approximately 15 minutes. After discharge to the ward, she developed complex auditory hallucinations in both ears, with incessant repetition of Carmen’s Habanera aria that lasted for approximately ten days.

**Figure 2:**
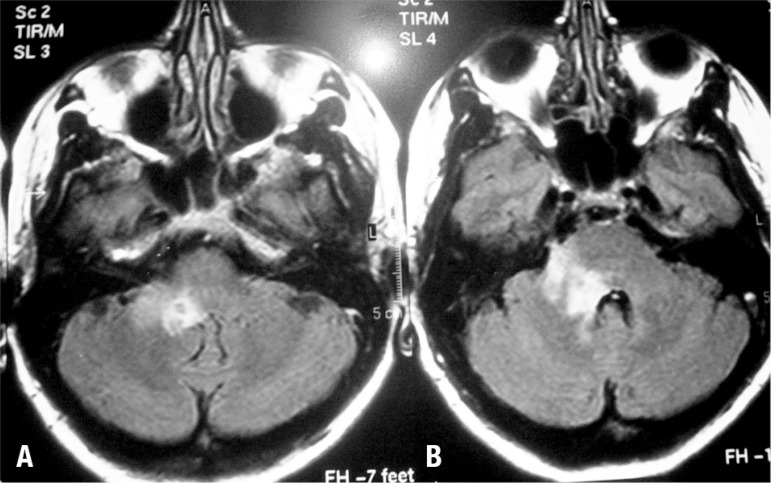
Recovering lesions after 21 days of treatment.

Treatment with intravenous acyclovir was discontinued after 21 days. The patient was discharged from hospital with continued neurological improvement. CSF still disclosed 39 cells (lymphomononuclear cells). She was maintained on oral acyclovir (2400 mg per day) for an additional 14 days. On the 50^th^ day into the disease, a repeat CSF exam showed one cell, glucose of 55 mg/dl and protein of 23 mg/dl. Follow up MRI showed recovering lesions (Figure 2) Flair weighted images demonstrated a reduction in the lesion in the right cerebellar peduncle and pons.

## Discussion

This case describes severe neurological impairment caused by brainstem encephalitis, with uncommon symptoms of bilateral auditory (AH) and visual hallucinations (VH), in the absence of other cognitive and behavioral symptoms, possibly caused by herpes encephalitis.

Hallucinations were initially described as cerebral-cortical-elicited phenomena in the late 19th century. In 1922, J. Lhermitte^4^ reported a case of a patient who presented VH after a presumed brainstem stroke.^1^ He later published five additional cases of VH, AH and tactile hallucinations.^5^ With these reports he coined the term “peduncular hallucinosis”, associated with tegmental midbrain lesions.^1^

AH may present in several conditions, such as alcoholism, exogenous intoxication, migraine, epilepsy, psychiatric disorders, inner ear diseases, sleep disturbances, delirium, as well as in pontine tegmentum, lower midbrain and temporal lobe lesions.^1,3,6,7^ Our patient did not have a history of illegal substance use, alcohol abuse, or a history of hearing loss. AH occurred in the setting of an acute brainstem insult. Hearing loss-related AH usually presents with somewhat different features, such as unilateral hallucination in the affected ear, that do not disappear over time. This kind of AH can be associated with visual and tactile hallucinations.^1,3^ AH associated with peripheral hearing loss are usually musical and affect the ears bilaterally especially in patients who become totally deaf.^8^

Anatomic lesions interrupting brainstem ascending and descending auditory pathways may also cause AH. Signals are generated by the cochlea, cochlear nuclei and superior olivary nuclei. The crucial lesion was therefore above these structures, at the level of the lateral lemniscus and the inferior colliculus. In unilateral ear involvement, symptoms are probably due to interruption of central fiber connections from the ventral and dorsal cochlear nuclei along the higher relay stations in the auditory pathway.^1^ One possible explanation for auditory hallucination with brainstem lesions is a “release phenomenon”, i.e. decreased sensory input perception leading to release of perceptual traces. A combination of peripheral and central disinhibition may cause this type of hallucination.^8^

Our patient also reported VH, at one point seeing the TV and furniture upside down. This type of VH is also known as oblique vision,^9^ usually associated with disturbed otolythic input into the thalamic nuclei. In our case, this symptom was probably related to a pontine lesion, involving ascending vestibular pathways (Deiters’ tract) from the brainstem vestibular to the vestibulothalamic nuclei.^10,11^

This case further illustrates a rare presentation of uncommon forms of visual and auditory hallucinations related to brainstem lesions. Although a more complete understanding of the underlying mechanisms involved in this rare and bizarre symptoms cannot be fully elucidated with isolated case reports, this case report serves to illustrates the nature and clinical course of these types of hallucinations and also allows speculation as to possible lesion sites involved in generating these uncommon symptoms.
